# Internal validation and improvement of mitochondrial genome sequencing using the Precision ID mtDNA Whole Genome Panel

**DOI:** 10.1007/s00414-021-02686-w

**Published:** 2021-09-07

**Authors:** Christian Faccinetto, Daniele Sabbatini, Patrizia Serventi, Martina Rigato, Cecilia Salvoro, Gianluca Casamassima, Gianluca Margiotta, Sara De Fanti, Stefania Sarno, Nicola Staiti, Donata Luiselli, Alberto Marino, Giovanni Vazza

**Affiliations:** 1 Reparto Carabinieri Investigazioni Scientifiche Di Parma, Sezione Biologia, Parma, Italy; 2 Department of Neurosciences DNS, University of Padova, Padova, Italy; 3 Department of Biology, University of Padova, Padova, Italy; 4 Department of Biological Geological and Environmental Sciences, University of Bologna, Bologna, Italy; 5 Interdepartmental Centre Alma Mater Research Institute On Global Challenges and Climate Change (Alma Climate), University of Bologna, Bologna, Italy; 6 Department of Cultural Heritage, University of Bologna, Ravenna, Italy

**Keywords:** Whole mitochondrial genome, Massive parallel sequencing, NGS, Internal validation, Ion Torrent, Forensic genetics

## Abstract

With the recent advances in next-generation sequencing (NGS), mitochondrial whole-genome sequencing has begun to be applied to the field of the forensic biology as an alternative to the traditional Sanger-type sequencing (STS). However, experimental workflows, commercial solutions, and output data analysis must be strictly validated before being implemented into the forensic laboratory. In this study, we performed an internal validation for an NGS-based typing of the entire mitochondrial genome using the Precision ID mtDNA Whole Genome Panel (Thermo Fisher Scientific) on the Ion S5 sequencer (Thermo Fisher Scientific). Concordance, repeatability, reproducibility, sensitivity, and heteroplasmy detection analyses were assessed using the 2800 M and 9947A standard control DNA as well as typical casework specimens, and results were compared with conventional Sanger sequencing and another NGS sequencer in a different laboratory. We discuss the strengths and limitations of this approach, highlighting some issues regarding noise thresholds and heteroplasmy detection, and suggesting solutions to mitigate these effects and improve overall data interpretation. Results confirmed that the Precision ID Whole mtDNA Genome Panel is highly reproducible and sensitive, yielding useful full mitochondrial DNA sequences also from challenging DNA specimens, thus providing further support for its use in forensic practice.

**Supplementary Information:**

The online version contains supplementary material available at 10.1007/s00414-021-02686-w.

## Introduction

Portions of the nuclear genome, such as autosomal short tandem repeat (STR) loci, are more often utilized during forensic investigations, as their typing results are much more informative than mitochondrial DNA (mtDNA). However, STR analysis on degraded samples remains highly controversial, mainly due to the difficulty of reliably interpreting results. Nonetheless, the lack of recombination, the abundance in cells, the matrilineal inheritance, and the apparently lower sensitivity to degradation make the mtDNA molecule highly suitable for forensic genetic investigations, especially for the analyses of bones, hairs shafts, teeth, and highly degraded biological samples [1].

So far, mtDNA analysis has been mainly based on the Sanger-type sequencing (STS) of the hypervariable segments (HVR-I, HVR-II) of the control region (CR), consisting of about 1100 bp with high polymorphic information content [2]. More recently, however, the development of robust sequencing protocols, the growth of high-quality databases, and the publication of guidelines for typing, annotating, and interpreting results from the International Society for Forensic Genetics allowed the scientific community to validate and rationalize the analysis of mtDNA in forensic casework [3, 4].

The recent advances in next-generation sequencing (NGS) technologies have generated an increasing interest in the use of mtDNA in forensic sciences, because of the potential capability of NGS to capture variants along the entire mitochondrial genome and to detect heteroplasmy at very low levels [5–7].

Among the recently developed NGS platforms, Illumina and Ion Torrent are the most used in forensic laboratories currently. Both platforms have been coupled with several typing kits and software extensions for sequencing and analysing the control region and the entire mitochondrial genome, reporting in general good performances in terms of reproducibility, specificity, and sensitivity when tested for casework implementation [8–12].

Despite these results, still further data and experience need to be accumulated to optimize technical and analytical protocols, and fully understand how these methods behave toward some biological and non-biological mtDNA features, as for example low-level heteroplasmy, mixture/contamination, and primer-binding site polymorphisms.

In this work, we describe the internal validation study for the NGS-based typing of the mtDNA genome, conducted using the Precision ID mtDNA Whole Genome Panel v.2.2 (Thermo Fisher Scientific, Waltham, MA, USA) on the Ion S5 system (Thermo Fisher Scientific), in accordance with the Validation Guidelines for Forensic DNA Analysis Methods of the Scientific Working Group on DNA Analysis Methods (*SWGDAM*) [13] and the European Network of Forensic Science Institute (ENFSI, Recommended Minimum Criteria for the Validation of Various Aspects of the DNA Profiling Process) [14].

## Materials and methods

### Sample description

The selected samples, analysed at the Reparto Carabinieri Investigazioni Scientifiche di Parma—RIS (Italy), were divided into a series of experiments in order to assess concordance, repeatability, reproducibility, sensitivity, and casework performance of the Precision ID mtDNA Whole Genome Panel (Table S1).

Concordance was determined in 6 replicates of each of the forensic standard control DNA samples 9947A (Thermo Fisher Scientific) and 2800 M (Promega; Madison, WI, USA), analysed using 0.1 ng of genomic DNA (gDNA) according to manufacturer’s recommendations. In addition, 8 saliva samples (DB4523, DB4538, DB4553, DB4579, DB4582, DB4588, DB4595, and DB4597), collected from anonymous volunteers after informed consent, were analysed using 0.1 ng of input gDNA and compared with their mtDNA control regions (CR) obtained at RIS by conventional Sanger-type sequencing (STS) (see Supplemental material for STS protocol).

An inter-laboratory concordance study was also performed by comparing NGS data produced with the Ion S5 system (Thermo Fisher Scientific) at RIS with those produced with an Ion PGM™ System (Thermo Fisher Scientific) at the Laboratory of Molecular Anthropology (Department of Biological, Geological and Environmental Sciences—BiGeA, University of Bologna, Italy). Two single-source samples from buccal swabs (BO08 and BO09), obtained from voluntary adults following written informed consent, were analysed at RIS, using manufacturer’s recommended 0.1 ng input gDNA, and at BiGeA, following the protocol described in De Fanti and collaborators [15].

The precision and accuracy of the Precision ID mtDNA Whole Genome Panel were assessed with two experiments. First, a repeatability study was performed using 0.1 ng of input gDNA of forensic standard control 9947A, analysed in 6 replicates by the same scientist on the same Ion S5 System. Second, a reproducibility study was performed using 0.1 ng of input gDNA of control samples 9947A and 2800 M; each examined in triplicate by two different scientists on the same Ion S5 System.

For sensitivity studies, 13 serial dilutions were manually prepared in UltraPure™ DNase/RNase-Free Distilled Water (Thermo Fisher Scientific) using the 9947Acontrol DNA, and examined in triplicate at the final gDNA input of 0.1 ng (X.1), 20 pg (X.2), 10 pg (X.3), 5 pg (X.4), 2.5 pg (X.5), 1.2 pg (X.6), 0.6 pg (X.7), 0.3 pg (X.8), 0.15 pg (X.9), 0.075 pg (X.10), 0.0375 pg (X.11), 0.01875 pg (X.12), and 0.009375 pg (X.13).

The casework study sample set consisted of 6 challenging DNA specimens (6 hairs shafts) analysed using ≤ 0.1 ng input gDNA, and their putative reference samples (1 post-mortem blood and 1 buccal swab) at the final gDNA input of 0.1 ng.

### DNA extraction and quantification

Since mtDNA typing is extremely sensitive to contamination, in both the RIS and BiGeA laboratories, all pre-amplification analyses were performed in a dedicated mtDNA laboratory, physically and logistically isolated from post-PCR facilities, following rigorous laboratory measures commonly used for ancient DNA analysis [16].

Saliva samples were collected with the Oragene DNA (OG-500) kit (DNA Genotek; Ottawa, Ontario, Canada) and extracted at BiGeA with the prepIT-L2P kit (DNA Genotek). Human buccal cell samples for the inter-laboratory concordance study were extracted at BiGeA with the QIAamp® DNA Mini Kit (Qiagen GmbH; Hilden, Germany) following manufacturer’s protocol.

Casework hair shaft samples were previously cleaned with different washing steps (10% Sodium hypochlorite, UltraPure™ DNase/RNase-Free Distilled Water, and 70% Ethanol), and DNA was extracted at the RIS laboratory using the Tissue and Hair Extraction Kit combined with the DNA IQ™ System (Promega) following manufacturer’s protocols for mitochondrial DNA isolation. Finally, the DNA of the putative reference samples of the casework specimens were isolated at the RIS using the EZ1® DNA Investigator ® Kit (Qiagen) with BioRobot EZ1 system (Qiagen) [17].

The amount of gDNA was determined in duplicate using the Quantifiler™ Trio DNA Quantification Kit (Thermo Fisher Scientific) on an Applied Biosystems 7500 Fast Real-Time PCR instrument and the HID Real-Time PCR Software v 1.2 (Thermo Fisher Scientific). DNA samples were then diluted in UltraPure™ DNase/RNase-Free Distilled Water to achieve 0.1 ng of final DNA, except for the sensitivity and casework study samples, where different concentrations were prepared as described above.

### Library and template preparation

Library preparation was performed using the Precision ID mtDNA Whole Genome Panel and the Ampliseq™ Precision ID Library Kit 2.0 (Thermo Fisher Scientific) according to manufacturer’s user guide (revision B.0, 2019) for a “two-in-one” method. This panel, specifically developed for forensic applications, consists in a 2-pool AmpliSeq multiplex assay of 81 primer pairs (average amplicon length of 162 bp with amplicon overlap of 11 bp), and a large number of additional degenerate primers (~ 280) to ensure full amplicon coverage with extremely degraded specimens [10, 18, 19].

Both extraction negative and library negative controls were included and processed identically to positive samples, to monitor the absence of contamination throughout the laboratory process. Furthermore, the 9947A forensic standard control DNA was used as a positive amplification control and a library preparation control during the experimental steps.

After primer digestion and adapter ligation, all the libraries were quantified using the Ion Library TaqMan™ Quantification Kit (Thermo Fisher Scientific) following manufacturer’s instructions and normalized to 30 pM in order to ensure an equal representation of each library in the pool. Samples with less than the desired 30 pM library concentration were used undiluted for library pooling. The barcoded libraries were combined (5 µl of each), and the resulting pool was quantified in triplicate to verify that the previous library normalization step was performed correctly. Fully automated template preparation, enrichment of template-containing beads, and chip loading were performed by the Ion Chef™ System (Thermo Fisher Scientific) using the Ion S5™ Precision ID Chef Kit (Thermo Fisher Scientific), according to manufacturer’s recommendations (ThermoFisher Scientific Application Guide: Precision ID mtDNA Panels with the HID Ion S5™/HID Ion GeneStudio™ S5 System, Revision B.0; January 19, 2019).

### Sequencing and data analysis

All samples analysed in this study were processed in five sequencing runs on an Ion S5™ System using the Ion S5™ Precision ID Sequencing Kit and the Ion 520™ sequencing chips (Thermo Fisher Scientific) following manufacturer’s instructions. Primary sequencing data were obtained using the Torrent Suite™ Software (TSS) v.5.10 (Thermo Fisher Scientific) and aligned to the revised Cambridge reference sequence (rCRS + 80) (NCBI reference NC_012920) [20] plus a repetition of 80 nucleotides after position 16,569 (as provided by Thermo Fisher Scientific) with default alignment options. Secondary sequencing analyses were performed with the HID Genotyper v.2.1 plug-in with Converge Software v.2.1 (both Thermo Fisher Scientific). Converge uses “mito variant caller” (MVC) [21], an optimized Smith-Waterman alignment algorithm [22] that integrates PhyloTree mtDNA phylogeny (http://www.phylotree.org) [23] and EMPOP (http://www.empop.org) [24] information into the scoring function. The MVC parameters were set as default: a minimum total read coverage per position of 20 reads, a minimum variant coverage of 20 reads to call, a coverage threshold to mark a region of 20 reads, and a minimum coverage percent compared to the median of the amplicon of 5.0. Additional MVC parameters included the following thresholds: 96.0 for confirming variant calls, 10.0 for point heteroplasmy (PHP), 20.0 for insertion, and 30.0 for deletion. The results concerning variant, coverage, and quality score were generated automatically by Converge Software v.2.1 in tabular format and linear-circular plots. Read alignments from the mvc.BAM (binary alignment map) and mvc.BAI (binary alignment index) files were manually inspected by two different scientists to confirm variants and anomalies, using both Integrative Genomes Viewer v.2.4.16 (IGV) [25] and the mitoIGV tool available in Converge. Mitochondrial haplogroups provided by Converge were confirmed using Haplogrep2 [26], a web server based on PhyloTree, Built 17. Data handling was performed with custom Python scripts, statistical analyses were done in R (version 4.0.2), and graphics were generated using the ggplot2 R package (version 2.2.1).

## Results and discussion

### Analysis of amplification-negatives

**Fig. 1:**
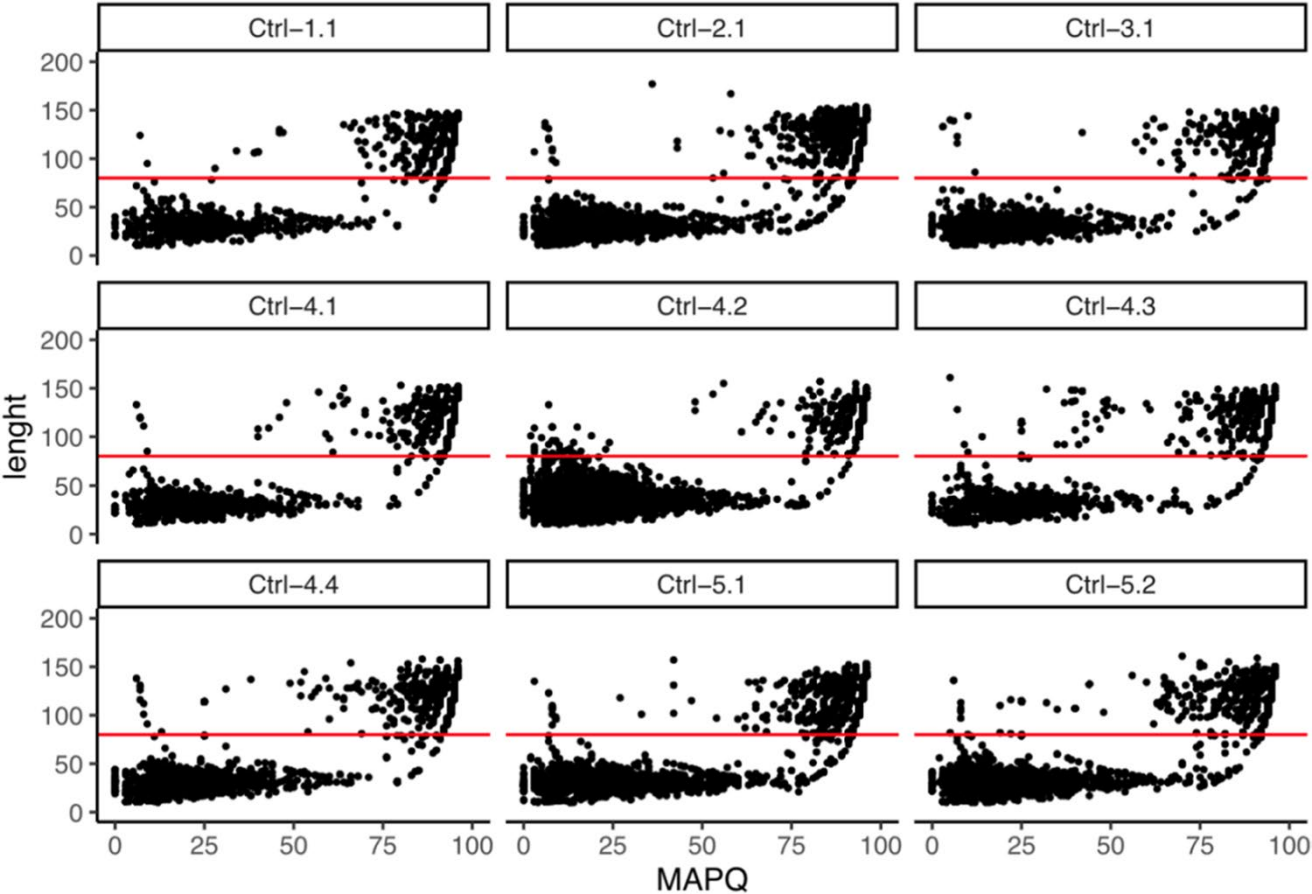
Mapped reads in amplification negatives. The scatterplots show the distribution of read length vs MAPQ in each amplification negative. The red line indicates the length cut-off value of 80 bp

As previously reported, it is well known that NGS methods tend to produce not negligible levels of background noise in sequencing results. This can be due both to technical artefacts during library preparation and target amplification (i.e. cytosine deamination, guanine oxidation) and to exogenous contamination of reagents, consumables or the laboratory environment [27–29]. To assess the potential impact of background noise, we analysed results from 9 amplification negatives sequenced throughout the study. Negative controls were distributed across the 5 runs as follows: one per run for runs 1, 2, and 3; four in run 4; and two in run 5. None of the 9 negative controls provided a complete mtDNA sequence, nor usable data for comparison; however, all of them exhibited few amplicons covered by aligned reads with a local maximum coverage ranging from 77 to 657. A deeper investigation of these reads clearly showed that most of them (mean of 69.05%, 95% CI 47.15–90.95%) were noticeably short reads (Table 1) with poor mapping quality (MAPQ) scores, as reported in Fig. 1. Conversely, long reads with high-quality alignment were a small proportion of the signal in all negatives.

**Table 1:** Short reads in amplification negatives

Sample	Total mapped reads	Reads < 80 bp	% of reads < 80 bp
Ctrl-1.1	2972	1762	59.2
Ctrl-2.1	7244	3958	54.6
Ctrl-3.1	3049	2094	69.0
Ctrl-4.1	2829	1807	63.8
Ctrl-4.2	6301	5494	87.1
Ctrl-4.3	2963	1852	62.0
Ctrl-4.4	6386	4982	78.0
Ctrl-5.1	8764	6334	72.2
Ctrl-5.2	6949	5247	75.5

Despite the origin of these reads being unclear, they can result at least in part from non-target micro-amplicons, created by the forward primer of one targeted amplicon and the reverse primer of a second, overlapping amplicon, or from very low levels of contaminating DNA. Regardless, these reads seem shorter than all the amplicons of the Precision ID mtDNA Whole Genome Panel whose expected size ranges from 125 to 174 bp. To reduce the noise associated with these short reads, we performed a cluster analysis of reads based on their length and MAPQ. The analysis identified the coordinates of the centroids of 2 clusters, of short low-quality and long high-quality reads, with an average of midpoints of 74.16 bp for length (95% CI 70.62–79.7) and of 57.6 for MAPQ (95% CI 55.95–59.27). We thus defined a length cut-off threshold of 80 bp and removed sequences < 80 bp by setting this filter in Torrent Suite ™ Software before alignment of reads and variant calling. We also evaluated the possibility to include a MAPQ threshold for the filtering, which would likely provide more accurate results, but unfortunately, the MAPQ score was not easily manageable in Torrent Suite ™ Software. A similar approach, based on read length only, has been effectively adopted by Michael D. Brandhagen and colleagues for the validation of another NGS-based mitochondrial DNA analysis, with the PowerSeq™ CRM Nested System (Promega) on the Illumina MiSeq platform [12]. Further, our read filtering step successfully removed the noise due to short sequences, providing an average read depth per position in negative controls of 9.4 reads (standard deviation of 5.9).

Interestingly, the removed short reads were not randomly distributed across the mtDNA but mainly occurred in the mt_125, mt_139, mt_164, mt_130, and mt_133 amplicons (also considering different runs), thus suggesting that they may be technical artefacts due to the formation of micro-amplicon sequences (Table S2). In contrast, longer reads left after filtering displayed a random distribution in negative controls, between and within different runs.

### Concordance study

Concordance was assessed by (i) comparing standard control DNAs with their known reference mtDNA profiles (6 replicates of control DNAs 9947A and 2800 M) [12, 30, 31], (ii) comparing sequences from 8 saliva samples to their previously obtained STS sequences of the CRs mtDNA (DB4523, DB4538, DB4553, DB4579, DB4582, DB4588, DB4595, and DB4597), and (iii) comparing whole mtGenomes sequenced in two laboratories with different Ion Torrent-based sequencing platforms (BO08 and BO09 samples) (Table S1).

The analysis of all the samples followed the same procedure as above including the short-read filtering (< 80 bp) to reduce noise. Although in these samples short reads were on average a small fraction of all mapped reads (2.70%, 95% CI 1.88–3.5%), they showed a non-homogeneous distribution similar to that seen in negative controls, and they could thus impact variant calling and heteroplasmy interpretation.

Across the 22 samples, the median value of the total number of mapped reads per sample was 202,312 (interquartile range [IQR], 183,269–241,234) with an average of 233,399. The median of the average base coverage per sample was 1383 reads ([IQR] 1246–1650), with an average of 96.87% uniformity of base coverage. The average amplicon coverage per sample had a median value across the samples of 1254 reads ([IQR] 1135–1495) with an average of 1448 reads (Table S3).

The entire mtDNA sequence was obtained for all the samples, except for two saliva specimens (DB4523 and DB4588), each presenting gaps on their mtDNA sequence due to low coverage amplicons. In sample DB4523, a fragment of 33 bp (nucleotide position (np) 13,216–13,248, margins included) and one of 69 bp (np 13,248–13,316) were missed in amplicon mt_129. Likewise, in sample DB4588, a gap of 91 bp spanning from amplicons mt_12 to mt_13 was observed at np 10,062–10,152. Moreover, a lower quality and filtering scores were observed around the poly-C stretches np 303–310 (HVS-II) and np 16,184–16,193 (HVS-I), possibly because of an inaccurate flow-call and pH fluctuation in the proton-based system. Also, a specific region comprising amplicons mt_79 to mt_80 (np 8248–8256) was found to be affected by extreme overall read strand bias which could be the result of amplification or alignment errors or contamination by nuclear mitochondrial DNA (NUMT) [19]. To assess the potential impact of the short reads filtering on the coverage of these regions, we reanalysed the samples without applying the read size cut-off. No substantial improvement in coverage of these regions was observed.

Overall, a total of 459 sequence variants were identified by NGS. Fully concordant mtDNA profiles were obtained for all the replicates of the 2800 M control DNA, as well as for the control region of the 8 samples previously analysed by Sanger sequencing. Some discrepancies were instead observed in the profiles of two samples: BO08 and 9947A. In the BO08 sample, which was analysed in two different labs by different NGS protocols and platforms (see “Sample description” in “Materials and methods”), multiple insertions at the homopolymeric regions 513–525 (524.AC) were detected by the Ion S5 (85.6% variant frequency) but not by the Ion PGM. STS based-typing of the CRs (see STS protocol in supplementary data), confirmed the presence of the AC repeat variant.

In all the replicates of the 9947A control DNA, the haplotypes were concordant with the NGS data described in Riman et al. [30], except for two of the three known point heteroplasmies (PHP). Although PHP at position 7861Y (T/C) was correctly reported in all the 9947A replicates (mean variant frequency 17.1%), the other two known heteroplasmic sites at positions 1393R (G/A) and 3242R (G/A) were never identified with the 10% default threshold. Again, to rule out the potential impact of the short-read filtering on the identification of these PHP, we reanalysed the samples without this filtering step and we obtained the same results. The differences in PHPs observed in 9947A control DNA are described below. With the mtDNA sequence variants detected, a phylogenetic check was performed in HaploGrep2; haplogroups are reported in Table S4. The mtDNA haplogroup composition of the 12 different samples (and replicates) revealed a typical Western European example of Eurasian haplogroups. Indeed, the samples analysed were assigned to 6 different mtDNA lineages and sub-lineages belonging to the H, HV, R, T, U, and X macro-haplogroups (Table S4).

### Repeatability and reproducibility

Repeatability and reproducibility of the PrecisionID mtDNA Whole Genome Panel were evaluated by comparing sequencing results from libraries prepared by a single scientist from identical samples on the same Ion S5 instrument, and by two different scientists on the same Ion S5 platform (Table S1 and S3). The depth of coverage pattern was similar among all the replicates: the average base coverage across samples was 2507 reads (median, 2224; [IQR] 1650–2766, with an average of 96.31% uniformity of base coverage), the average amplicon coverage across the samples was 2752 (median, 2034; [IQR] 1495–2853) reads, and the mean number of mapped reads across all the samples was 368,202 (median 329,142; [IQR] 241,234–403,842) (Table S3). The haplotype calls for the 18 full mtDNA were completely concordant with the reported 9947A and 2800 M sequences [12, 30, 31] except for the above mentioned PHPs 1393R and 3242R in 9947A replicates.

### Case type study

Forensic specimens that contain few copies of DNA by their nature, such as hair shafts, human skeletal remains, or highly degraded samples, remain a challenge to the forensic DNA typing community. In order to evaluate the performance of the Precision ID mtDNA Whole Genome Panel in typical forensic specimens, a total of 6 hair shafts (9–1, 24–1, 24–2, 30–2, 51–2, and 51–3) were analysed twice in two different runs with their putative reference samples (49–28 blood sample and 53–1 buccal swab). All those samples were previously subjected to STR analysis by conventional CE-based typing and sequenced at their mtDNA CRs using STS. Full STR profiles were obtained only for the two reference samples, while from STS analysis, partial HVS-I sequences were obtained for 5 hair shafts (9–1, 24–1, 24–2, 51–2, 51–3). Using NGS, hair samples 30–2 and 51–3 yielded very partial mtGenome sequences with large uncovered regions, possibly because of the severely degraded mtDNA. Therefore, these two samples were excluded from further interpretation results. Full mtDNA sequences were obtained for the two reference samples (49–28, 53–1) and for four (9–1, 24–1, 24–2, 51–2) of the six hair shaft samples. For these latter four casework samples, the median of average base coverage in NGS data was 1254 reads ([IQR] 1142–1607), the median of average amplicon coverage was 1182 ([IQR] 1075–1507) reads, while the median of total number of mapped reads was 188,089 ([IQR] 173,292–241,014) (Table S5).

MtDNA sequence obtained via the Precision ID mtDNA Whole Genome Panel was consistent with the available partial HVS-I Sanger data for each sample (Table S4). In addition, an identical mtDNA (haplogroup N9a1) was obtained from hair samples 24–1, 24–2, and 51–2, and the reference sample 49–28 (blood). Analogously, hair shaft 9–1 showed the same haplotype (haplogroup D4e1a1) of the reference sample 53–1 (buccal swab), indicating a complete concordance of the results obtained with the two methods and that the Precision ID mtDNA Whole Genome Panel is fit for the purpose to use with typical forensic specimens.

### Analysis of variant frequency distribution

Previous studies suggested that the haplotype purity (i.e. the number and percentage of reads supporting a sequence variant) may be diagnostic in ascertaining the correctness of that variant call [8]. High levels of noise due to PCR artefacts, alignment errors, or NUMTs can, therefore, complicate the analysis, leading to false-positive or false-negative calls, as well as false heteroplasmy detection.

**Fig. 2:**
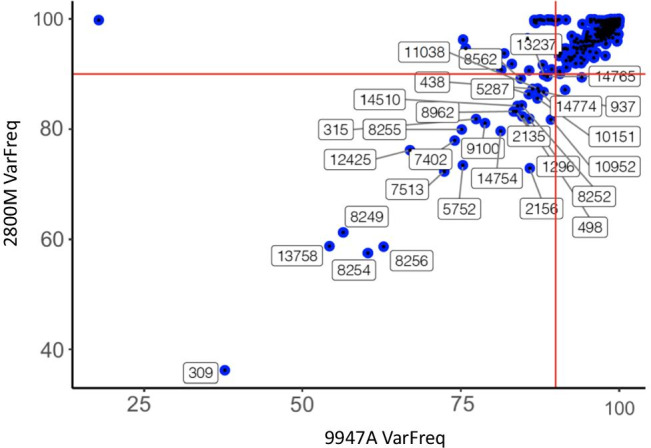
The scatterplot shows the mean VarFreq of each positions across all the replicates of 9947A (*x* axis) and 2800 M (*y* axis). The red line indicates a VarFreq of 90% (see text). Positions with a VarFreq below 90% in both controls are labelled

The distribution of variant frequencies (VarFreq) in the 9947A and 2800 M standard controls was then analysed and compared within- and between-run replicates used for the concordance study. No significant differences in the overall VarFreq distribution were observed within and between runs (6 replicates in 2 runs for each control DNA) thus confirming the reproducibility of the system. The empirical cumulative distribution function of VarFreq was computed considering all replicates divided into dilution series to obtain the proportion of positions ≤ VarFreq in a range of 0–99 VarFreq. Of the 16,648 mtDNA positions, 503 (3%) showed a VarFreq < 99% and 53 (0.3%) < 90%. The positions with VarFreq < 90% showed a remarkable recurrence in multiple replicates of the same sample as well as across different samples. In particular, some of them (i.e. positions 310 and 16,189) are associated to *C-stretch* variations, and the difficulties in variant calling within homopolymer stretches have already been reported for Ion systems [8, 30]. Further positions corresponded to known NUMTs and artefact sequences reported in previous studies, such as region 8248–8256 np [19, 32] while others showed a consistent noise level across samples and runs (Fig. 2; Table 2). However, it is known that NUMTs are more apt to be co-amplified by short-amplicon PCR approaches, like the ion technique, in respect of the long-amplicon PCR [33]. Despite neither false-positives nor negative calls being identified in our samples, the recurrent noise at these positions could be the source of erroneous calls and PHP detection; therefore, variants in these positions should be interpreted with caution.

**Table 2:** Positions with VarFreq < 90% across multiple replicates of 9947A and 2800 M of samples

Position	rCRS base	Sample	VarFreq mean	VarFreq standard deviation	Second most common variant
309*	C	9947A	37.76	23.81	DEL
		2800 M	36.18	5.69	
315	C	9947A	77.40	10.21	INS
		2800 M	81.84	4.08	
438	C	9947A	86.36	6.83	DEL
		2800 M	87.33	4.34	
498	C	9947A	84.75	2.13	DEL
		2800 M	82.32	2.74	
937	T	9947A	88.09	6.60	DEL
		2800 M	86.77	3.67	
1296*	A	9947A	85.84	6.01	DEL
		2800 M	81.91	5.73	
2135	A	9947A	83.75	4.54	DEL
		2800 M	83.21	5.95	
5287*	A	9947A	85.76	6.11	DEL
		2800 M	86.34	1.48	
5752*	A	9947A	75.32	14.18	DEL
		2800 M	73.43	9.89	
7402	C	9947A	74.03	6.31	DEL
		2800 M	77.93	9.17	
7513	T	9947A	72.39	13.67	DEL
		2800 M	72.28	9.02	
8249	G	9947A	56.47	14.13	INS
		2800 M	61.26	4.32	
8252	C	9947A	84.66	4.62	G
		2800 M	84.34	5.54	
8254	C	9947A	60.31	5.32	DEL
		2800 M	57.51	5.16	
8255*	G	9947A	75.14	11.57	DEL
		2800 M	79.94	4.71	
8256	T	9947A	62.78	10.49	DEL
		2800 M	58.67	5.08	
8962	A	9947A	83.35	5.73	DEL
		2800 M	83.22	5.42	
9100	A	9947A	78.83	4.36	G
		2800 M	81.09	3.85	
10,151	A	9947A	87.12	5.91	DEL
		2800 M	85.59	4.16	
11,038	A	9947A	87.17	5.73	DEL
		2800 M	87.41	3.10	
12,425	A	9947A	66.95	12.42	DEL
		2800 M	76.17	4.26	
13,237*	A	9947A	88.15	4.95	DEL
		2800 M	89.89	2.88	
13,758	C	9947A	54.32	6.28	DEL
		2800 M	58.76	4.84	
14,510	A	9947A	83.97	7.57	DEL
		2800 M	84.23	5.51	
14,754*	C	9947A	81.30	12.77	DEL
		2800 M	79.64	5.44	
14,774*	C	9947A	87.38	10.76	DEL
		2800 M	86.89	3.94	

*NUMT positions reported in Li et al. [32]

### Sensitivity study

Sensitivity of the Precision ID mtDNA Whole Genome Panel was tested using the 9947A forensic standard control with different input amounts of gDNA ranging from 100 to 0.009375 pg (Table S1). Each DNA quantity was amplified in triplicate using the same PCR cycle conditions (24 PCR cycles). The results of the sensitivity study on the samples are summarized in Table 3. Full mtDNA coverage (100%) was successfully achieved for all the replicates of 9947A down to 0.15 pg input gDNA (X9 dilution), except for one replica at 10 pg (X3 dilution), which resulted with a loss of about 39% of the mtDNA sequence probably due to pipetting errors during amplification. Down to 0.6 pg (X7), all the replicates covering full mtDNA matched the known 9947A mtDNA sequence, with the exception of the two PHP positions mentioned above (1393R and 3242R). For the remaining dilutions with full DNA coverage, 0.3 pg (X8) and 0.15 pg (X9) input gDNA, in one out of the three replicates the expected 7861Y heteroplasmy was also missed. PCR replicates from X10 (0.075 pg) to X13 (0.009375 pg) showed stochastic variation and several gaps on mtDNA with a coverage ranging from 47.3 to 96.7%. Sequencing results of these replicates showed evident stochastic variation (with drops of expected calls and introduction of false-positive calls) when less than 0.15 pg of gDNA was used as PCR input.

**Table 3:** Proportion of mtDNA sequenced for dilution series

Sample	X1	X2	X3	X4	X5	X6	X7	X8	X9	X10	X11	X12	X13
	100%	100%	100%	100%	100%	100%	100%	100%^#^	100%	90.8%*	96.6%*	80.7%*^§^	72.4%*
*9947A*	100%	100%	61.5%*	100%	100%	100%	100%	100%	100%^#^	91.7%*	63.4%*	91.6%*	47.3%*^§^
	100%	100%	100%	100%	100%	100%	100%	100%	100%	93.6%*	96.7%*	62.4%*^§^	49.9%*^§^

^*^Incomplete mtDNA profile.

^**#**^Undetected 7861Y heteroplasmy.

^§^Presence of false positive variant calls.

**Fig. 3:**
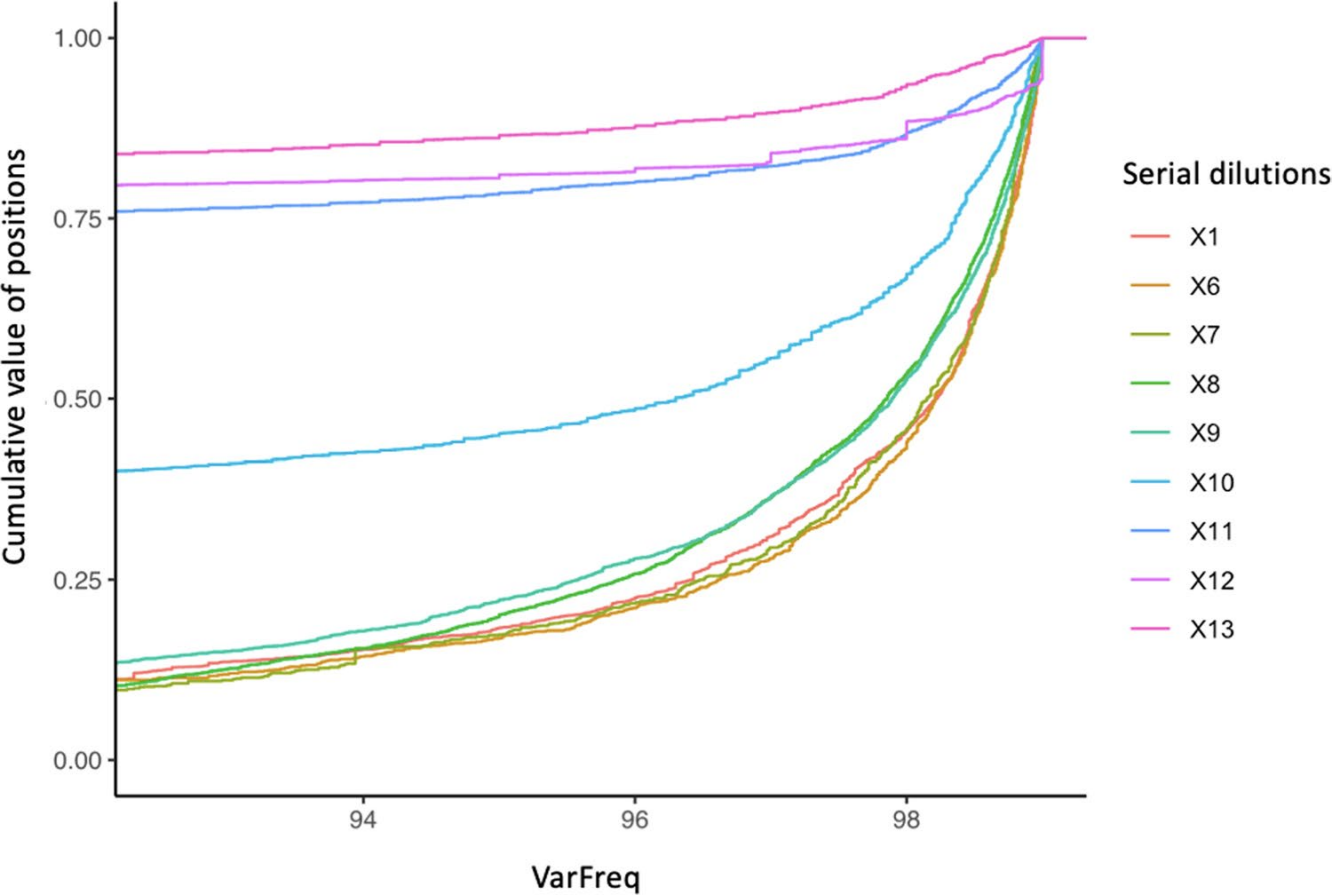
Cumulative VarFreq distribution computed in the range of 0–99% of each dilution series (considering all replicates). For a better interpretation, the 92.5–99% VarFreq range is shown

Interestingly, the analysis of the cumulative VarFreq distribution of these dilution series reflected the obtained results with clear differences between likely stochastic and non-stochastic outputs (Fig. 3). In fact, all replicates with a good outcome (from X1 to X7) showed a similar VarFreq distribution, while all the replicates with false negative and/or false positive calls (from X8 to X13) displayed a decrease of VarFreq in many positions which corresponds to a loss of haplotype purity. This data could be used to have an immediate picture of the overall quality of the sequencing data. Indeed, independently from the quantitation of mtDNA or gDNA, the cumulative VarFreq distribution could be a good parameter in assessing whether a sequencing run is reliable. However, further studies with larger sample sizes would be needed to validate and implement this approach.

Overall, the sensitivity study using the 9947A control DNA demonstrated that the Precision ID mtDNA Whole Genome Panel provided full and reliable mtDNA sequences down to 0.6 pg, although we have to consider the lot-to-lot variability in the cell lines used as controls [34]. Using the standard equation provided by the Thermo Fisher Scientific protocol (0.1 ng of gDNA ~ 2900 copies of mtDNA), 0.6 pg of gDNA would contain about 17 copies of mtDNA, clearly that is a rather unrealistic approximation. A specific mtDNA quantification would be required to identify the usage limit of the Precision ID mtDNA Whole Genome Panel in terms of the mtDNA copy number.

### Heteroplasmy

**Fig. 4:**
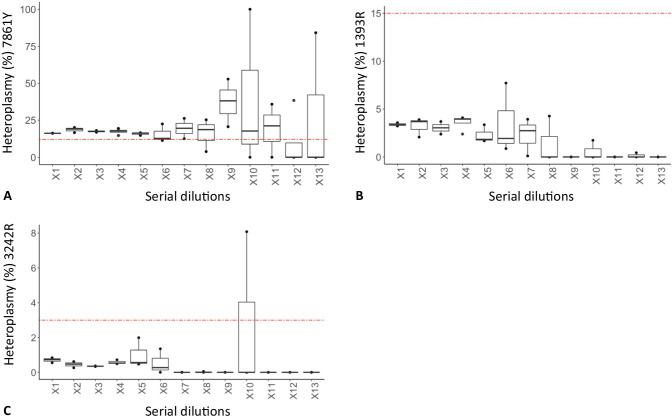
The plot shows the values of point heteroplasmies in the 9947A dilution series. **A** PHP 7861Y (T/C); **B** PHP 1393R (G/A); **C** PHP 3242R (G/A). The dashed red line indicates the expected heteroplasmy value

Point heteroplasmy detection was assessed in both the high-quantity replicates and the dilution replicates of the sensitivity study of the 9947A control DNA. As previously mentioned, when a standard 10% VarFreq cut-off was applied, only PHP 7861Y (T/C) was correctly called in all the high-quantity replicates. In this case, we obtained a median heteroplasmy of 17.1% ± 1.2% slightly higher than the one reported by Riman et al. (about 12%) [30]. The same PHP was correctly called in all the replicates of the dilution series up to X7. However, as reported in Fig. 4A, starting from the X6 dilution, the heteroplasmy quantification became more variable as gDNA input decreased up to a completely random detection and quantification in the most extreme dilutions (X9-X13). On the contrary, the 1393R PHP, expected at a frequency of about 15%, was never called either in high-quantity replicates (median 3.2% ± 0.7%) or in all the dilution series (Fig. 4B). Of note, the short-read filtering step did not affect the frequency estimate of this PHP. As suggested in recent studies, a possible explanation of these differences may be related to the NGS platform, chemistry, software setting, and the lot-to-lot variability of the control DNAs [30, 35]. Similarly, the low-level heteroplasmy at site 3242, expected at a frequency of about 3%, showed a VarFreq far below 1% in all high-input DNA replicates (median 0.7% ± 0.1%) as well as in most of the dilution series (Fig. 4C). Lowering the heteroplasmy cut-off to correctly call these low-level PHPs produced a number of false positives, as expected from the previous analysis of the VarFreq distribution of all the mtDNA calls.

## Conclusions

We here describe the evaluation of Precision ID Whole mtDNA Genome Panel performed on an Ion S5 platform using control DNAs 2800 M and 9947A as well as typical forensic specimens.

A preliminary analysis of negatives highlighted the presence of a non-negligible level of background noise related to the alignment of very short reads. The introduction of a filtering step for reads > 80 bp in the analytical workflow greatly reduced the noise level and improved the accuracy of variant calling. The analysis of VarFreq along the entire mtGenome highlighted the presence of positions with a systematic low VarFreq across different runs and samples. Based on this information, variants and heteroplasmy calls in these positions require special attention and should be carefully considered by the analysts. Regarding PHP detection, the system has the potential to be extremely sensitive; however, the VarFreq distribution makes it difficult to distinguish between real and false calls when low PHP are considered. Further bioinformatic solutions are desirable to improve overall PHP interpretation. From this perspective, the use of a probabilistic approach based on the analysis of the VarFreq distribution would allow the estimation of probabilities for each PHP call and set a more appropriate PHP threshold for each sample based on sequencing results. Overall, validation experiments confirmed that the Precision ID Whole mtDNA Genome Panel is able to generate accurate, reproducible, and reliable whole mitochondrial genome sequences and is more sensitive than STS on casework forensic samples.

## Supplementary Information

Below is the link to the electronic supplementary material.

Supplementary file1 (DOCX 63 KB)

## Data Availability

The data that support the findings of this study are available from the corresponding authors, G.V. and C.F., upon reasonable request.
