# Supplementary material for: Internal validation and improvement of mitochondrial genome sequencing using the Precision ID mtDNA Whole Genome Panel
Source: Int J Legal Med. 2021 Sep 7;135(6):2295–306. doi: 10.1007/s00414-021-02686-w (PMC8523450; doi:10.1007/s00414-021-02686-w)
Supplement: Supplementary file 1 — Supplementary file1 (DOCX 63 KB) [file 414_2021_2686_MOESM1_ESM.docx]

**Table S1:** Description of the analyzed samples

|  | **Sample** | **Sample origin** | **gDNAinput**  **(pg)** | **Replicates** | **Data comparison** |
| --- | --- | --- | --- | --- | --- |
| *Concordance* | 9947A | control DNA | 100 | 6 | NIST whole mtDNA reference sequence* |
|  | 2800M | control DNA | 100 | 6 | NIST whole mtDNA reference sequence* |
|  | DB4523 | saliva | 100 | 1 | Sanger sequencing of the control region |
|  | DB4538 | saliva | 100 | 1 | Sanger sequencing of the control region |
|  | DB4553 | saliva | 100 | 1 | Sanger sequencing of the control region |
|  | DB4579 | saliva | 100 | 1 | Sanger sequencing of the control region |
|  | DB4582 | saliva | 100 | 1 | Sanger sequencing of the control region |
|  | DB4588 | saliva | 100 | 1 | Sanger sequencing of the control region |
|  | DB4595 | saliva | 100 | 1 | Sanger sequencing of the control region |
|  | DB4597 | saliva | 100 | 1 | Sanger sequencing of the control region |
|  | BO08 | buccal swab | 100 | 1 | Inter-laboratory concordance, whole mtDNA ** |
|  | BO09 | buccal swab | 100 | 1 | Inter-laboratory concordance, whole mtDNA ** |
| *Repeatability* | 9947A | control DNA | 100 | 6 replicates | whole mtDNA |
| *Reproducibility* | 9947A | control DNA | 100 | 3 replicates (operator 1)  3 replicates (operator 2) | whole mtDNA |
|  | 2800M | control DNA | 100 | 3 replicates (operator 1) 3 replicates (operator 2) | whole mtDNA |
| *Sensitivity* | 9947A_X1 | control DNA | 100 | 3 | whole mtDNA |
|  | 9947A_X2 | control DNA | 20 | 3 | whole mtDNA |
|  | 9947A_X3 | control DNA | 10 | 3 | whole mtDNA |
|  | 9947A_X4 | control DNA | 5 | 3 | whole mtDNA |
|  | 9947A_X5 | control DNA | 2.5 | 3 | whole mtDNA |
|  | 9947A_X6 | control DNA | 1.2 | 3 | whole mtDNA |
|  | 9947A_X7 | control DNA | 0.6 | 3 | whole mtDNA |
|  | 9947A_X8 | control DNA | 0.3 | 3 | whole mtDNA |
|  | 9947A_X9 | control DNA | 0.15 | 3 | whole mtDNA |
|  | 9947A_X10 | control DNA | 0.075 | 3 | whole mtDNA |
|  | 9947A_X11 | control DNA | 0.0375 | 3 | whole mtDNA |
|  | 9947A_X12 | control DNA | 0.01875 | 3 | whole mtDNA |
|  | 9947A_X13 | control DNA | 0.00937 | 3 | whole mtDNA |

* Reference sequence from Brandhagen et al. [1]; Lee et al. [2]; Riman et al. [3]

** samples sequenced on an Ion PGM^TM^ System (Thermo Fisher Scientific) and reported in De Fanti et al. [4]

**Table S2:** Amplicons with highest proportion of short reads.

| **Amplicon number** | **Start-Stop positions** | **Observed**  **times** | **Mean of amplicon coverage** |
| --- | --- | --- | --- |
| 125 | 16034-16159 | 9/9 | 171.11 |
| 139 | 2166-2305 | 9/9 | 46.66 |
| 164 | 8522-8686 | 9/9 | 39.4 |
| 130 | 436-566 | 8/9 | 8.25 |
| 133 | 15037-15170 | 6/9 | 29.83 |

Observed times: number of times the amplicon resulted in the top 10 amplicons for highest short read coverage across the 9 negative controls.

**Table S3:** Summary of sequencing information per sample for concordance, repeatability and reproducibly analyses

| **Study** | **Sample ID** | **Chip** | **Mapped**  **reads** | **Average base coverage** | **Uniformity of base coverage** | **Average amplicon coverage** |
| --- | --- | --- | --- | --- | --- | --- |
| Repeatability | 9947A.1 | 1 | 692629 | 4683.76 | 74.87% | 4282 |
|  | 9947A.2 | 1 | 286119 | 1939.48 | 96.07% | 1767 |
|  | 9947A.3 | 1 | 223775 | 1533.77 | 98.51% | 1382 |
|  | 9947A.4 | 2 | 361011 | 2451.12 | 99.30% | 2231 |
|  | 9947A.5 | 2 | 380950 | 2618.79 | 99.45% | 2377 |
|  | 9947A.6 | 2 | 472518 | 3209.46 | 98.82% | 8591 |
| Concordance and Reproducibility | 9947A.1 | 3 | 288243 | 1973.31 | 97.12% | 1786 |
|  | 9947A.2 | 3 | 275881 | 1887.88 | 95.32% | 1700 |
|  | 9947A.3 | 3 | 136657 | 939.90 | 86.18% | 848 |
|  | 9947A.4 | 4 | 198443 | 1359.27 | 99.37% | 1230 |
|  | 9947A.5 | 4 | 202668 | 1388.24 | 99.37% | 1257 |
|  | 9947A.6 | 4 | 194270 | 1328.98 | 99.37% | 1203 |
| Concordance and Reproducibility | 2800M.1 | 3 | 297272 | 1997.57 | 98.13% | 1836 |
|  | 2800M.2 | 3 | 754188 | 5171.75 | 97.39% | 4678 |
|  | 2800M.3 | 3 | 371488 | 2521.81 | 97.13% | 2301 |
|  | 2800M.4 | 4 | 230937 | 1581.01 | 98.68% | 1432 |
|  | 2800M.5 | 4 | 244666 | 1673.32 | 98.68% | 1516 |
|  | 2800M.6 | 4 | 102871 | 698.68 | 98.68% | 637 |
| Concordance | DB4523 | 4 | 194769 | 1327.8 | 97.68% | 1212 |
|  | DB4538 | 4 | 139754 | 951.98 | 94.50% | 863 |
|  | DB4553 | 4 | 180068 | 1222.63 | 98.77% | 1115 |
|  | DB4579 | 4 | 207355 | 1414.36 | 99.37% | 1282 |
|  | DB4582 | 4 | 192872 | 1316.59 | 98.08% | 1196 |
|  | DB4588 | 4 | 112203 | 843.4 | 79.90% | 743 |
|  | DB4595 | 4 | 174226 | 1182.93 | 98.78% | 1079 |
|  | DB4597 | 4 | 201955 | 1377.62 | 99.37% | 1252 |
|  | B08 | 4 | 205167 | 1397 | 99.40% | 1267 |
|  | B09 | 4 | 228826 | 1563.63 | 99.97% | 1417 |

**Table S4:** List of mtDNA haplotypes motifs and related haplogrups from NGS analysis.

| Sample ID | Haplotypes (range 1-16569) | Haplogroup |
| --- | --- | --- |
| DB4523 | 60.1T 64T 263G 750G 1438G 2355G 2442C 2706G 3847C 4769G 7028T 8860G 9922T 12295C 13188T 13651G 14766T 15326G 15674C 16126C 16230G 16362C 16497G | R0a2k |
| DB4538 | 73G 153G 195C 225A 226C 263G 309.1C 315.1C 502T 750G 1438G 1719A 2706G 4769G 6221C 6371T 7028T 8256del 8269A 8393T 8860G 8950A 11719A 12705T 13708A 13966G 14470C 14766T 14818C 15326G 15927A 16189C 16223T 16278T 16519C | X2b3 |
| DB4553 | 263G 315.1C 750G 1438G 4769G 7269A 7278C 8093C 8860G 9804A 15326G 16220C 16274A 16292T 16318G 16519C | H |
| DB4579 | 55.1T 57C 59C 151T 152C 263G 309.1C 315.1C 709A 750G 930A 1438G 1888A 2706G 4216C 4769G 4917G 5147A 5656G 7028T 8697A 8860G 10463C 10750G 11251G 11719A 11812G 13368A 14233G 14766T 14905A 15326G 15452A 15607G 15928A 16126C 16292T 16294T 16296T 16304C 16519C | T2b3a1 |
| DB4582 | 55.1T 57C 59C 73G 151T 152C 189G 263G 315.1C 709A 750G 930A 1438G 1888A 2706G 4216C 4769G 4917G 5147A 5656G 7028T 8697A 8860G 10463C 10750G 11251G 11719A 11812G 13368A 14233G 14766T 14905A 15326G 15452A 15607G 15928A 16126C 16292T 16294T 16296T 16304C 16519C | T2b3a1 |
| DB4588 | 143A 263G 315.1C 750G 1438G 4769G 6776G 15326G 16519C | H3 |
| DB4595 | 73G 263G 310C 523del 524del 750G 1438G 1617T 1719A 2706G 4769G 6221C 6371T 6791G 7028T 8503C 8860G 11008T 11719A 11939T 12477C 12705T 13184C 13708A 13966G 14470C 14766T 15300C 15326G 16182C 16183C 16189C 16193.1C 16223T 16278T 16390A 16519C | X2d2 |
| DB4597 | 263G 309.1C 315.1C 750G 1438G 3010A 4769G 8860G 15326G 15499T 16042A 16356C 16362C 16519C | H1b |
| B08 | 73G 263G 309.1C 315.1C 455del 524.AC 750G 1438G 2706G 3197C 4769G 7028T 8860G 9477A 9548A 11087C 11467G 11719A 12308G 12372A 13617C 14684T 14766T 14793G 15326G 16168T 16192T 16256T 16270T 16304C 16526A | U5a2b3 |
| B09 | 263G 309.1C 315.1C 489C 750G 1438G 2706G 4769G 5902C 7028T 7094C 8860G 11087C 15326G 15514C 15924G 16192A 16519C | HV4 |
| 49-28 | 73G 150T 263G 309.1C 315.1C 750G 1438G 2706G 4386C 4769G 5231A 5417A 7028T 7830A 8860G 10586A 11719A 12007A 12358G 12372A 12705T 14766T 15326G 16111T 16129A 16223T 16257A 16261T 16293C | N9a1 |
| 53-1 | 73G 94A 194T 263G 309.1C 315.1C 489C 750G 1438G 2706G 3010A 3316A 4769G 4883T 5178A 5628C 5964C 7028T 8414T 8701G 8860G 9053A 9536T 9540C 10398G 10400T 10873C 11215T 11719A 12705T 14470C 14668T 14766T 14783C 15043A 15301A 15326G 15924G 16093C 16176T 16223T 16362C | D4e1a1 |
| 9-1 | 73G 94A 194T 263G 309.1C 315.1C 489C 750G 1438G 2706G 3010A 3316A 4769G 4883T 5178A 5628C 5964C 7028T 8414T 8701G 8860G 9053A 9536T 9540C 10398G 10400T 10873C 11215T 11719A 12705T 14470C 14668T 14766T 14783C 15043A 15301A 15326G 15924G 16093C 16176T 16223T 16362C | D4e1a1 |
| 24-1 | 73G 150T 263G 309.1C 315.1C 750G 1438G 2706G 4386C 4769G 5231A 5417A 7028T 7830A 8860G 10586A 11719A 12007A 12358G 12372A 12705T 14766T 15326G 16111T 16129A 16223T 16257A 16261T 16293C | N9a1 |
| 24-2 | 73G 150T 263G 309.1C 315.1C 750G 1438G 2706G 4386C 4769G 5231A 5417A 7028T 7830A 8860G 10586A 11719A 12007A 12358G 12372A 12705T 14766T 15326G 16111T 16129A 16223T 16257A 16261T 16293C | N9a1 |
| 51-2 | 73G 150T 263G 309.1C 315.1C 750G 1438G 2706G 4386C 4769G 5231A 5417A 7028T 7830A 8860G 10586A 11719A 12007A 12358G 12372A 12705T 14766T 15326G 16111T 16129A 16223T 16257A 16261T 16293C | N9a1 |
| 9947A | 93G 195C 214G 263G 309.CC 315.1C 750G 1438G 4135C 4769G 7645C 7861Y 8448C 8860G 9315C 13572C 13759A 15326G 16311C 16519C | H11b1 |
| 2800M | 152C 263G 315.1C 477C 750G 1438G 3010A 4769G 8860G 15326G 16519C | H1c |

**Table S5:** Summary sequencing information for case-type samples

| **Sample ID** | **Mapped**  **reads** | **Average base coverage** | **Uniformity of base coverage** | **Average amplicon coverage** |
| --- | --- | --- | --- | --- |
| 49-28 | 323357 | 2201,05 | 99,56% | 1997 |
| 53-1 | 236284 | 322,57 | 99,37% | 1462 |
| 9-1.1 | 75076 | 494,26 | 98,85% | 466 |
| 9-1.2 | 181754 | 1199,32 | 96,12% | 1127 |
| 24-1.1 | 184066 | 1233,46 | 98,67% | 1167 |
| 24-1.2 | 282337 | 1883 | 99,29% | 1790 |
| 24-2.1 | 227239 | 1515,42 | 98,79% | 1413 |
| 24-2.2 | 380218 | 2510,24 | 97,20% | 2338 |
| 30-2.1 | 2719 | 17,78 | 38,02% | 16 |
| 30-2.2 | 1942 | 12,50 | 45,08% | 12 |
| 51-2.1 | 192112 | 1275,22 | 98,68% | 1197 |
| 51-2.2 | 147908 | 973 | 99,97% | 919 |
| 51-3.1 | 114983 | 695,60 | 53,22% | 684 |
| 51-3.2 | 749 | 4,83 | 73,83% | 4 |

**Sanger-type sequencing (STS) of the control region (CR)**

The mtDNA control region was amplified as five overlapping fragments (L15989-H16433; L16197-H16509; L16450-H180; L109-H460; L317-H599) [5] in order to obtain 1121 base pairs (bp) encompassing nucleotide position (np) from 16024 to 576. PCR amplification was carried out in a Veriti™ 96-Well Thermal Cycler (Applied BioSystems, Foster City, USA) in a final volume of 25 μL of reaction mix containing 1 µL DNA template, 2.5 µL 10 X AmpliTaq Gold-Buffer (Applied BioSystems), 2 µL MgCl_2_ (25 mM), 2.5 µL dNTP mix (10 mM), 0.5 µL primers FW and RV (10 µM), 0.25 µL AmpliTaq Gold DNA Polymerase (5U/µM) (Applied BioSystems), and 18.75 µL of nuclease-free water. The PCR thermal cycle conditions consisted of initial denaturation at 95°C for 11 min, 39 cycles of 15 sec at 95°C, 10 sec at 57°C and 20 sec at 72°C, followed by a final extension for 7 minutes at 72°C. Afterwards, PCR products were checked on 1.5% agarose gel and subsequently purified using ExoSAP-IT reagent (Applied BioSystems) following manufacturer’s recommendation. Sequencing reaction was performed in a final volume of 10 µL using BigDye Terminator sequencing reagent (Applied BioSystems, v3.1) and 10µM of each primer for sequencing reaction. PCR was performed in a Veriti™ 96-Well Thermal Cycler (Applied BioSystems) comprising an initial denaturation at 96°C for 2min and 10 sec, 30 cycles of 10 sec at 96°C, 15 sec at 50°C and extension for 4 sec at 60°C. Sequencing reaction products were purified from residual dye terminators using BigDye XTerminator Purification Kit (Applied BioSystems), according to the manufacturer’s user guide. Finally, mtDNA sequencing was carried out on an ABI Prism 3110xL Genetic Analyzer using POP 6, 50-cm capillary arrays (all Applied Biosystems), and default instrument settings as recommended by the manufacturer. To reduce ambiguities in sequence determination, the forward and reverse primers were used to sequence both strands of mtDNA control region. Extraction blanks, negative and positive controls were carried out through the entire amplification and sequencing processes. Obtained sequencing data were aligned and compared to the revised Cambridge Reference Sequence (rCRS, GenBank Accession Number NC 012920) [6] with BioEdit v7.2.5 [7] and MEGAX [8] software. Mitochondrial haplogroups were determined based on Haplogrep2 software [9] and the PhyloTree mtDNA phylogeny, built 17 (www.phylotree.org) [10].

**References:**

1. Brandhagen MD, Just RS, Irwin JA (2020) Validation of NGS for mitochondrial DNA casework at the FBI Laboratory. Forensic Sci Int Genet 44:102151. https://doi.org/10.1016/j.fsigen.2019.102151

2. Lee EY, Lee HY, Oh SY, et al (2016) Massively parallel sequencing of the entire control region and targeted coding region SNPs of degraded mtDNA using a simplified library preparation method. Forensic Sci Int Genet 22:37–43. https://doi.org/10.1016/j.fsigen.2016.01.014

3. Riman S, Kiesler KM, Borsuk LA, Vallone PM (2017) Characterization of NIST human mitochondrial DNA SRM-2392 and SRM-2392-I standard reference materials by next generation sequencing. Forensic Sci Int Genet 29:181–192. https://doi.org/10.1016/j.fsigen.2017.04.005

4. De Fanti S, Vianello D, Giuliani C, et al (2017) Massive parallel sequencing of human whole mitochondrial genomes with Ion Torrent technology: an optimized workflow for Anthropological and Population Genetics studies. Mitochondrial DNA Part A DNA Mapping, Seq Anal 28:843–850. https://doi.org/10.1080/24701394.2016.1197218

5. Berger C, Parson W (2009) Mini-midi-mito: Adapting the amplification and sequencing strategy of mtDNA to the degradation state of crime scene samples. Forensic Sci Int Genet 3:149–153. https://doi.org/10.1016/j.fsigen.2009.01.011

6. Andrews RM, Kubacka I, Chinnery PF, et al (1999) Reanalysis and revision of the Cambridge reference sequence for human mitochondrial DNA. Nat Genet 23:147–147. https://doi.org/10.1038/13779

7. Hall TA (1999) BIOEDIT: a user-friendly biological sequence alignment editor and analysis program for Windows 95/98/ NT. Nucleic Acids Symp Ser 41:95–98

8. Kumar S, Stecher G, Tamura K (2016) MEGA7: Molecular Evolutionary Genetics Analysis Version 7.0 for Bigger Datasets. Mol Biol Evol 33:1870–1874. https://doi.org/10.1093/molbev/msw054

9. Kloss-Brandstätter A, Pacher D, Schönherr S, et al (2011) HaploGrep: A fast and reliable algorithm for automatic classification of mitochondrial DNA haplogroups. Hum Mutat 32:25–32. https://doi.org/10.1002/humu.21382

10. van Oven M, Kayser M (2009) Updated comprehensive phylogenetic tree of global human mitochondrial DNA variation. Hum Mutat 30:E386–E394. https://doi.org/10.1002/humu.20921
